# The Analytical Solutions to a Cation–Water Coupled Multiphysics Model of IPMC Sensors

**DOI:** 10.3390/s26020695

**Published:** 2026-01-20

**Authors:** Kosetsu Ishikawa, Kinji Asaka, Zicai Zhu, Toshiki Hiruta, Kentaro Takagi

**Affiliations:** 1 Department of Mechanical Engineering, Toyohashi University of Technology, Toyohashi 441-8580, Japan; ishikawa.kosetsu.dx@tut.jp (K.I.); hiruta.toshiki.kp@tut.jp (T.H.); 2 Research Organization of Science and Technology, Ritsumeikan University, Kusatsu 525-8577, Japan; kar21133@fc.ritsumei.ac.jp; 3 Department of Mechanical Engineering, Xi’an Jiaotong University, Xi’an 710049, China; zicaizhu@xjtu.edu.cn

**Keywords:** ionic polymer–metal composite, modeling, transfer function, polymer sensor

## Abstract

Ionic polymer–metal composite (IPMC) sensors generate voltages or currents when subjected to deformation. The magnitude and time constant of the electrical response vary significantly with ambient humidity and water content. However, most conventional physical models focus solely on cation dynamics and do not consider water dynamics. In addition to cation dynamics, Zhu’s model explicitly incorporates the dynamics of water. Consequently, Zhu’s model is considered one of the most promising approaches for physical modeling of IPMC sensors. This paper presents exact analytical solutions to Zhu’s model of IPMC sensors for the first time. The derivation method transforms Zhu’s model into the frequency domain using Laplace transform-based analysis together with linear approximation, and subsequently solves it as a boundary value problem of a set of linear ordinary differential equations. The resulting solution is expressed as a transfer function. The input variable is the applied bending deformation, and the output variables include the open-circuit voltage or short-circuit current at the sensor terminals, as well as the distributions of cations, water molecules, and electric potential within the polymer. The obtained transfer functions are represented by irrational functions, which typically arise as solutions to a system of partial differential equations. Furthermore, this paper presents analytical approximations of the step response of the sensor voltage or current by approximating the obtained transfer functions. The steady-state and maximum values of the time response are derived from these analytical approximations. Additionally, the relaxation behavior of the sensor voltage is characterized by a key parameter newly derived from the analytical approximation presented in this paper.

## 1. Introduction

Ionic polymer–metal composites (IPMCs) are a category of electro-active polymers and can potentially be applied to soft robotics and wearable devices [1,2,3,4,5,6]. IPMCs are typically used as actuators; however, potential applications as sensors have also been reported [7,8]. Applications of IPMC sensors include wearable devices [9,10,11,12,13], energy harvesters [14,15], medical applications [16,17], and fluid measurements [18]. An IPMC consists of an ion-exchange polymer membrane sandwiched between noble metal electrodes, typically gold or platinum. The ion-exchange polymer, typically a cation-exchange resin such as Nafion, contains mobile counterions, which are cations in the case of Nafion, and a solvent, typically water, within the polymer. Under bending deformation of an IPMC, a pressure gradient is generated along the thickness direction. Subsequently, cations and water molecules migrate toward the region with lower water pressure, specifically toward the outer electrode. The redistribution of cations along the thickness direction generates an electric field between the two electrodes. Consequently, open-circuit voltage [1,7,19] or short-circuit current [20] can be measured. The open-circuit voltage is approximately proportional to the deformation magnitude but exhibits a dynamic response with slow relaxation [19,21]. The short-circuit current exhibits an approximately proportional relationship with the deformation speed and also demonstrates a dynamic response. Furthermore, the dynamic response depends on the polymer species, ionic species, and water content [19]. Therefore, physical models that can explain changes in the dynamic response of IPMCs due to variations in materials and environmental conditions must be established.

A wide range of modeling approaches has been explored for IPMCs, spanning from black-box to white-box (physics-based) models [22]. Black-box and gray-box models are typically constructed from experimental data using phenomenological or empirical methods. For IPMCs, for example, black-box models based on system identification have been reported [23,24,25], as well as gray-box models [26,27,28]. Although these models are effective in capturing input–output behavior, they often lack interpretability and generalizability beyond the experimental conditions under which they were developed. In contrast, the present study focuses on white-box models derived from first principles, with the objective of capturing the underlying physics governing the behavior of IPMCs. These physical models describe ion migration, electric potential, and mechanical deformation using systems of partial differential equations. In the early stages of research on modeling IPMC actuators, several models based on the thermodynamics of irreversible processes [29,30] and frictional effects [31,32] were proposed [33]. Over time, the Poisson–Nernst–Planck (PNP) framework has become the dominant approach for modeling both IPMC sensors and actuators. The PNP equations, which couple the Poisson equation with the Nernst–Planck equation, describe the electrodiffusion of ions in an electrostatic field and provide an effective framework for modeling ion transport within the polymer matrix. Nemat-Nasser [34] first applied this framework to IPMC actuators. Subsequently, numerous studies have adopted and extended the PNP approach. Notable examples for actuators include the works of Wallmersperger et al. [35,36,37], Pugal et al. [38,39], Porfiri [40], Cha et al. [41,42], and Chen et al. [43].

Regarding white-box modeling of IPMC sensors [44], Farinholt et al. [20] extended the PNP approach to sensor modeling. In recent years, the PNP-based framework has remained the mainstream approach for sensor modeling. Notable contributions include those by Chen et al. [45] and Aureli et al. [46]. Among these, Chen et al. [45] were the first to derive an analytical solution to a PNP-based IPMC sensor model. Their solution, expressed as an irrational transfer function, enabled analysis of the dynamic response of the system in the frequency domain. Aureli et al. [46] extended this approach by considering nonlinear ion transport and applied the matched asymptotic expansions method to obtain an approximate analytical solution in the time domain. These analytical treatments provided valuable insights into system dynamics and useful tools for sensor design and control implementation. However, most conventional physical models are limited in that they account only for cation dynamics and neglect water dynamics. Previous studies have shown that sensor responses are strongly influenced by humidity and internal water content, leading to significant variations in both response amplitude and time constant [19,47]. Therefore, to accurately capture sensor dynamics and guide model-based sensor design, it is essential to incorporate both cation and water dynamics into the modeling framework.

Physical models of IPMC sensors must account for variations in humidity and water content. Zhu proposed a model that explicitly considers water as a solvent [48,49]. In addition to the dynamics of cations within an IPMC, Zhu’s model incorporates the dynamics of water, an aspect that had not been considered in earlier studies. Following Zhu’s work, several models accounting for the dynamics of water molecules have been proposed [50,51,52]. Such models can be used to numerically simulate the response of an IPMC sensor as a function of humidity (or the water content of the polymer). To date, no studies have attempted to derive analytical solutions for Zhu’s model [49] or for alternative models [50,51,52]. The complexity of these models arises from systems of nonlinear partial differential equations, which make the derivation of analytical solutions challenging. As a result, previous research has primarily relied on numerical solutions using finite element software such as COMSOL [49,50,51,52]. An analytical method employing approximations and simplifications has also been proposed [53]; however, exact analytical solutions remain unclear.

This paper presents, to the best of our knowledge, exact analytical solutions for Zhu’s multiphysics model of IPMC sensors for the first time. Preliminary results related to the mathematical derivation of the analytical solution have been reported previously [54,55]. However, these studies are incomplete because they neither derive analytical solutions for the sensor current nor include physical considerations. Therefore, the present study provides exact analytical solutions for the sensor voltage, sensor current, and associated physical interpretations. The proposed approach involves several steps. First, Zhu’s model is linearly approximated and expressed in vector form. Next, the model is transformed into the frequency domain using Laplace transform-based analysis. It is then solved as a boundary-value problem for a set of linear ordinary differential equations. The resulting exact analytical solution is expressed as a transfer function matrix in the Laplace and spatial variables. The input to the transfer function matrix is the applied bending curvature, and the outputs include the open-circuit voltage or short-circuit current at the terminal electrodes, as well as the distributions of cations, water molecules, and electric potential within the polymer. The obtained transfer functions are represented by irrational functions, which typically arise as solutions to systems of partial differential equations. The frequency response is derived using these transfer functions. Furthermore, this paper presents analytical approximations of the step response of the sensor voltage and current by approximating the obtained transfer functions. The steady-state and maximum values of the time response are derived from these analytical approximations. Moreover, a key parameter that predicts the relaxation behavior of the sensor is identified. This parameter, which includes diffusion coefficients and concentration constants, determines the relaxation behavior of the sensor voltage. Consequently, the voltage relaxation behavior can be explained in terms of physical constants associated with cations, water, and the polymer.

## 2. Zhu’s Multiphysics Model of IPMC Sensors

### 2.1. Governing Equations

**Figure 1:**
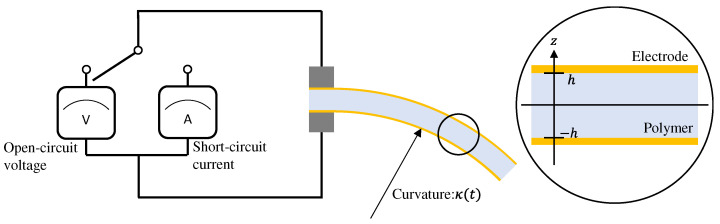
Schematic of an IPMC sensor.

In this study, we propose a model for the generated open-circuit voltage *V* or short-circuit current *I* when a beam-shaped IPMC sensor is subjected to a uniform curvature κ, as shown in Figure 1. The open-circuit voltage can be measured using a voltage amplifier with high input impedance, as shown in Figure 1. Hereinafter, this measurement condition is referred to as the “voltage-output case.” The short-circuit current can be measured using a transimpedance amplifier, which sets the electric potential difference between the two electrodes to zero, as shown in Figure 1. Hereinafter, this measurement condition is referred to as the “current-output case.”

An intuitive explanation of the physical principle of IPMC sensors is provided as follows [44,45,49]. Initially, deformation of the IPMC sensor generates a pressure gradient along the polymer film thickness, which drives the motion of water molecules and cations. As a result, the concentrations of cations and water molecules are redistributed. The redistributed cation concentration affects the electric potential distribution within the membrane, thereby generating a voltage or current between the electrodes.

The following is a brief description of Zhu’s multiphysics model [48,49]. Zhu’s multiphysics model describes the physics of an IPMC sensor using cation concentration, water concentration, and electric potential. Although Zhu’s model does not fully account for certain interfacial phenomena, such as the dimensionality of the system (considering only one-dimensional space instead of the inherently three-dimensional structure), specific ion adsorption at interfaces, and the roughness of the polymer–metal interface, it is still capable of qualitatively simulating the sensor voltage and current responses. The variables used in the model are listed in Table 1, and the constants are listed in Table 2. As shown in Figure 1, the coordinate in the thickness direction of the IPMC sensor is denoted by *z*, the polymer boundaries are located at z=±h, and time is denoted by *t*. The pressure *P* within the polymer membrane varies with the curvature κ of the IPMC sensor, where κ denotes the externally applied bending. The pressure *P* is also affected by the local volume fraction of water $w_{V}$:

(1) $$P=\frac{E_{\mathrm{dry}}}{3}\left[{\left(\frac{1+w_{V}}{1+w_{V0}}\right)}^{-\frac{4}{3}}-{\left(\frac{w_{V}}{w_{V0}}\right)}^{-\frac{4}{3}}\right]-\frac{E_{\mathrm{wet}}}{3}z\kappa +\sigma^{*},$$

(2) $$w_{V}\left(c_{W}\right)=\frac{c_{W}}{\frac{\rho_{H_{2}O}}{M_{H_{2}O}}-c_{W}},$$

where $\sigma^{*}$ denotes the effects of osmotic pressure, electrostatic stress, and capillary pressure [48]. Equations (1) and (2) are based on the macroscopic model presented in [48,49], which was extended from Nemat-Nasser’s micromechanics model [34]. The effective elastic modulus of the wet polymer, $E_{\mathrm{wet}}$, is lower than that of the dry polymer, $E_{\mathrm{dry}}$, as stiffness is reduced by hydration [34].

The pressure *P* produces a flux of cations in the polymer, $J_{I}$, and a flux of water molecules, $J_{W}$:

(3) $$\begin{array}{ccc} J_{I} & = & -d_{II}\left(\frac{\partial c_{I}}{\partial z}+\frac{z_{I}c_{I}F}{R_{\mathrm{gas}}T}\frac{\partial \varphi }{\partial z}\right)-\frac{c_{I}}{c_{W}}n_{dW}d_{II}\frac{\partial c_{W}}{\partial z}-c_{I}K\frac{\partial P}{\partial z}, \end{array}$$

(4) $$\begin{array}{ccc} J_{W} & = & -n_{dW}d_{II}\left(\frac{\partial c_{I}}{\partial z}+\frac{z_{I}c_{I}F}{R_{\mathrm{gas}}T}\frac{\partial \varphi }{\partial z}\right)-d_{WW}\frac{\partial c_{W}}{\partial z}-c_{W}K\frac{\partial P}{\partial z}. \end{array}$$

The following equation holds based on mass conservation:

(5) $$\frac{\partial c}{\partial t}+\frac{\partial J}{\partial z}=0,$$

where

(6) $$c:=\left[\begin{array}{c} c_{I}\\ c_{W} \end{array}\right],\;J:=\left[\begin{array}{c} J_{I}\\ J_{W} \end{array}\right].$$

Equations (3)–(5) are the key equations of Zhu’s multiphysics model and describe the dynamics of cations and water molecules. In general, the relationship among flux, concentration gradient of cations moving in an electrolyte, and electric potential gradient is described by the Nernst–Planck equation [34,48]. Equations (3) and (4) account for the dynamics of water molecules in addition to those of cations. Therefore, Zhu’s physical model represents an extended form of the Nernst–Planck equation. Assuming that there is no inflow or outflow of cations and water from the polymer to the electrodes, the boundary condition for J is obtained as follows:

(7) $${\left.J\right|}_{z=\pm h}=0.$$

The relationship between cation concentration and electric potential within the polymer is described by the Poisson equation as follows:

(8) $$\begin{array}{c} \frac{\partial }{\partial z}\left(\varepsilon \frac{\partial \varphi }{\partial z}\right)=-z_{I}F\left(c_{I}-c^{-}\right), \end{array}$$

where ε is the effective permittivity, and $\varepsilon =\varepsilon_{r}\varepsilon_{0}$.

**Figure 2:**
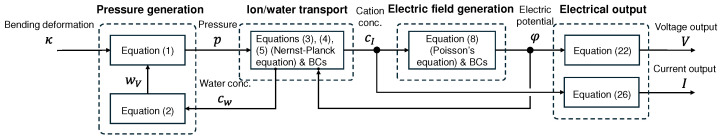
Block diagram of the multiphysics model of IPMC sensors.

A set of Equations (1)–(8) constitutes Zhu’s multiphysics model of IPMC sensors. This model describes how pressure is generated in an IPMC due to applied curvature deformation, leading to the motion of cations and water molecules, and how an electrical response arises from the resulting redistribution of cations. Figure 2 shows a block diagram illustrating the flow from the input to the output in Zhu’s model.

### 2.2. Linearized Governing Equations

In this study, Zhu’s model is linearized to derive exact analytical solutions. For IPMC sensors, previous studies [53], have demonstrated that the linear model effectively approximates the nonlinear model. In particular, COMSOL simulations reported in [53] showed that even under large deformations corresponding to a curvature of 450 m^−1^, which corresponds to a radius of curvature of approximately 2.2 mm, the error introduced by linearization remains within 0.1%.

$J_{I}$ and $J_{W}$ in Equations (3) and (4) are linearly approximated around the equilibrium point, respectively, as follows:

(9) $$\begin{array}{c} J_{I}\approx -d_{II}\frac{\partial c_{I}}{\partial z}-\frac{c_{I0}}{c_{W0}}\left(n_{dW}d_{II}+\frac{4}{9}KE_{\mathrm{dry}}\right)\frac{\partial c_{W}}{\partial z}-d_{II}\frac{z_{I}c_{I0}F}{R_{\mathrm{gas}}T}\frac{\partial \varphi }{\partial z}+\frac{c_{I0}KE_{\mathrm{wet}}}{3}\kappa , \end{array}$$

(10) $$\begin{array}{c} J_{W}\approx -n_{dW}d_{II}\frac{\partial c_{I}}{\partial z}-\left(d_{WW}+\frac{4}{9}KE_{\mathrm{dry}}\right)\frac{\partial c_{W}}{\partial z}-n_{dW}d_{II}\frac{z_{I}c_{I0}F}{R_{\mathrm{gas}}T}\frac{\partial \varphi }{\partial z}+\frac{c_{W0}KE_{\mathrm{wet}}}{3}\kappa . \end{array}$$

Here, $\sigma^{*}$ is neglected, because, although $\sigma^{*}$ it may influence the equilibrium potential, its effect on the transport process is negligible within the present approximation [49].

Substituting Equations (9) and (10) into Equation (5), and using Equation (8), the following equations are obtained:

(11) $$\frac{\partial c_{\Delta }}{\partial t}=D\frac{\partial^{2}c_{\Delta }}{\partial z^{2}}-Fc_{\Delta },$$

(12) $$\frac{\partial^{2}\varphi_{\Delta }}{\partial z^{2}}+\frac{z_{I}F}{\varepsilon }\left[\begin{array}{cc} 1 & 0 \end{array}\right]c_{\Delta }=0,$$

where

(13) $$c_{\Delta }=\left[\begin{array}{c} c_{I\Delta }\\ c_{W\Delta } \end{array}\right]:=\left[\begin{array}{c} c_{I}-c_{I0}\\ c_{W}-c_{W0} \end{array}\right],$$

(14) $$\begin{array}{ccc} \varphi_{\Delta } & := & \varphi -\varphi_{0}, \end{array}$$

(15) $$D:=\left[\begin{array}{cc} d_{II} & \frac{c_{I0}}{c_{W0}}\left(n_{dW}d_{II}+\frac{4}{9}KE_{\mathrm{dry}}\right)\\ n_{dW}d_{II} & d_{WW}+\frac{4}{9}KE_{\mathrm{dry}} \end{array}\right],$$

(16) $$\delta :=\frac{1}{z_{I}Fh}\sqrt{\frac{\varepsilon R_{\mathrm{gas}}T}{c_{I0}}},$$

(17) $$F:=\frac{1}{\delta^{2}h^{2}}D\left[\begin{array}{cc} 1 & 0\\ 0 & 0 \end{array}\right],$$

where $c_{I0}$(=c^−^), $c_{W0}\left(=\rho_{H_{2}O}w_{V0}/\left\{M_{H_{2}O}\left(1+w_{V0}\right)\right\}\right)$, and $\varphi_{0}$(=0) are the cation concentration, water concentration, and electric potential at equilibrium, respectively. Furthermore, δ is a dimensionless parameter known as small $\delta \approx 10^{-5}$ at room temperature [19,56].

### 2.3. Boundary Conditions and Output Equation for the Voltage-Output Case

If the open-circuit voltage is measured, the boundary conditions are given as follows.

(18) $${\left.\frac{\partial \varphi_{\Delta }}{\partial z}\right|}_{z=\pm h}=0.$$

Additionally, a suitable boundary condition for the electric potential is imposed to avoid an indefinite constant in the electric potential.

(19) $${\left.\varphi_{\Delta }\right|}_{z=0}=0.$$

Substituting the boundary conditions in Equations (7) and (18) into Equations (9) and (10), the boundary conditions in $c_{\Delta }$ can be obtained as:

(20) $$D{\left.\frac{\partial c_{\Delta }}{\partial z}\right|}_{z=\pm h}=b\kappa ,$$

where b is the constant vector defined as

(21) $$b:=\frac{KE_{\mathrm{wet}}}{3}\left[\begin{array}{c} c_{I0}\\ c_{W0} \end{array}\right].$$

The output equation for the voltage-output case is expressed as follows:

(22) $$V\left(t\right)={\left.\varphi_{\Delta }\right|}_{z=h}-{\left.\varphi_{\Delta }\right|}_{z=-h}$$

where *V* denotes the open-circuit voltage.

### 2.4. Boundary Conditions and Output Equation for Current-Output Case

If the short-circuit current is measured, the boundary conditions are given as follows:

(23) $${\left.\varphi_{\Delta }\right|}_{z=\pm h}=0.$$

Substituting Equations (7) and (23) into Equations (9) and (10), respectively, the boundary conditions on $c_{\Delta }$ can be obtained:

(24) $$D{\left.\frac{\partial c_{\Delta }}{\partial z}\right|}_{z=\pm h}=b\kappa -\frac{\varepsilon }{z_{I}F\delta^{2}h^{2}}D\left[\begin{array}{c} 1\\ 0 \end{array}\right]{\left.\frac{\partial \varphi_{\Delta }}{\partial z}\right|}_{z=\pm h}.$$

The output equation for the current-output case is expressed as follows:

(25) $$\begin{array}{c} I\left(t\right)=\frac{z_{I}FS}{2h}\int_{-h}^{h}\frac{\partial c_{I}}{\partial t}zdz=\frac{z_{I}FS}{2h}\int_{-h}^{h}J_{I}dz \end{array}$$

where *I* denotes the short-circuit current. Equation (25) can be rewritten [53] as follows:

(26) $$\begin{array}{c} I\left(t\right)=-z_{I}FS\left[\begin{array}{cc} 1 & 0 \end{array}\right]\left\{\frac{1}{2h}D\left({\left.c_{\Delta }\right|}_{z=h}-{\left.c_{\Delta }\right|}_{z=-h}\right)-b\kappa \right\}. \end{array}$$

### 2.5. Brief Experimental Validations

**Figure 3:**
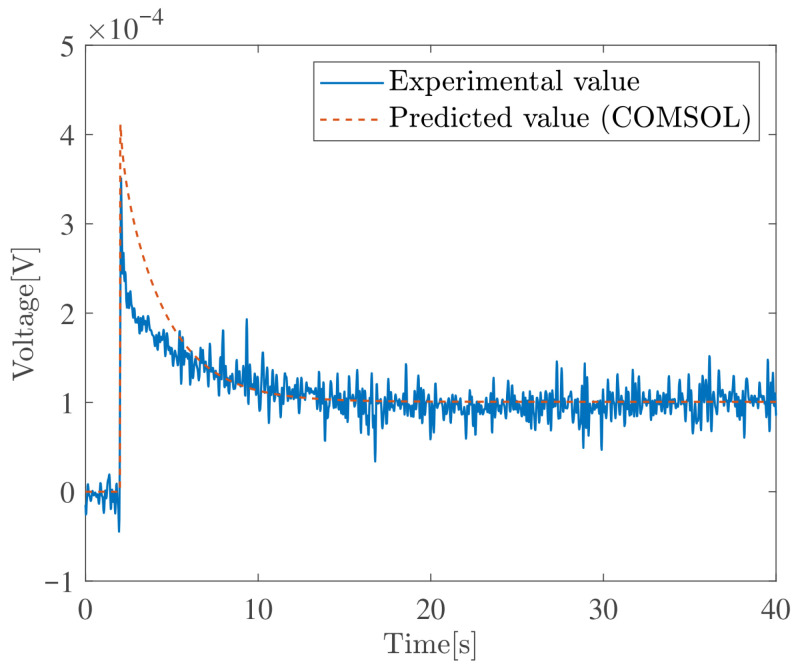
Validation of Zhu’s model. Sensor voltage response (2 mm step bending) of an Au–IPMC with ${\mathrm{Li}}^{+}$–cations at RH 90%.

**Figure 4:**
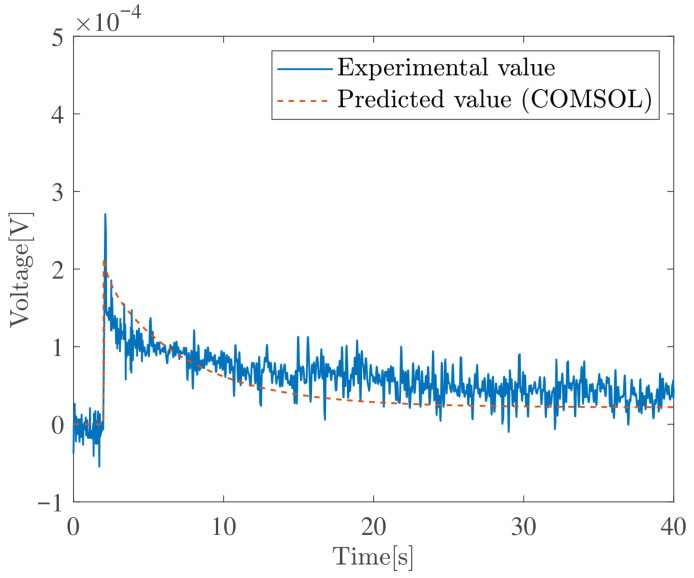
Validation of Zhu’s model. Sensor voltage response (2 mm step bending) of an Au–IPMCs with ${\mathrm{Na}}^{+}$–cations at RH 90%.

This section compares Zhu’s model with experimental results reported in [49]. The step response experimental data presented in [49] were used, and the model simulations were performed using COMSOL Multiphysics 6.4. The simulation constants were taken from Table 1.3 in [49]. Figure 3 shows the results for Au-IPMC with ${\mathrm{Li}}^{+}$ as the cation, and Figure 4 shows the results for Au-IPMC with ${\mathrm{Na}}^{+}$ as the cation. In the case of ${\mathrm{Li}}^{+}$, a finite steady-state voltage remains. In contrast, in the case of ${\mathrm{Na}}^{+}$, the sensor voltage relaxes to nearly zero even under continuous applied deformation.

These results indicate that Zhu’s model not only reproduces the relaxation behavior of the sensor voltage observed in experiments, but also captures whether a finite steady-state voltage remains, depending on the physical constants.

## 3. Exact Analytical Solutions in the Frequency Domain and Transfer Functions

### 3.1. General Solution

This subsection derives the general solution of Equations (11) and (12). First, Equations (11) and (12) are Laplace transformed with respect to time to obtain ordinary differential equations in *z*. The general solution is then obtained by solving the resulting set of ordinary differential equations. To begin, Equation (11) is Laplace transformed with respect to time as:

(27) $$s{\tilde{c}}_{\Delta }-{\left.c_{\Delta }\right|}_{t=0}=D\frac{\partial^{2}{\tilde{c}}_{\Delta }}{\partial z^{2}}-F{\tilde{c}}_{\Delta },$$

where ${\tilde{c}}_{\Delta }$ and ${\tilde{\varphi }}_{\Delta }$ are Laplace transforms of $c_{\Delta }$ and $\varphi_{\Delta }$, respectively. To derive the transfer function, we assume that the initial conditions are in equilibrium ${\left.c_{\Delta }\right|}_{t=0}=0$. Note that Equations (29) and (27) are ordinary differential equations for *z* and can be rewritten as follows:

(28) $$\frac{d^{2}{\tilde{c}}_{\Delta }}{dz^{2}}=\frac{1}{h^{2}}M\left(s\right){\tilde{c}}_{\Delta },$$

(29) $$\frac{d^{2}{\tilde{\varphi }}_{\Delta }}{dz^{2}}=-\frac{z_{I}F}{\varepsilon }\left[\begin{array}{cc} 1 & 0 \end{array}\right]{\tilde{c}}_{\Delta },$$

where

(30) $$M\left(s\right):=D^{-1}h^{2}s+\frac{1}{\delta^{2}}\left[\begin{array}{cc} 1 & 0\\ 0 & 0 \end{array}\right],$$

where D is assumed to be regular. The general solution of Equation (28) is then derived [54], as follows:

(31) $${\tilde{c}}_{\Delta }\left(z,s\right)=\sinh\left(N\left(s\right)\frac{z}{h}\right)q_{1}\left(s\right),$$

(32) $${\tilde{\varphi }}_{\Delta }\left(z,s\right)=-\frac{z_{I}Fh^{2}}{\varepsilon }\left[\begin{array}{cc} 1 & 0 \end{array}\right]M{\left(s\right)}^{-1}\sinh\left(N\left(s\right)\frac{z}{h}\right)q_{1}\left(s\right)+q_{3}\left(s\right)z,$$

where $q_{1}\left(s\right)$ and $q_{3}\left(s\right)$ are arbitrary functions of *s*. N(s) denotes the principal square root matrix of M(s) ($N\left(s\right)=\sqrt{M(s)}$). If M(s) is diagonalizable, N(s) is expressed as follows [57]:

(33) $$N=\frac{1}{\sqrt{\mathrm{tr}\left(M\right)+2\sqrt{\det(M)}}}\left(M+\sqrt{\det(M)}I_{2}\right),$$

where $I_{2}$ denotes the identity matrix of 2×2. The arbitrary functions $q_{1}\left(s\right)$ and $q_{3}\left(s\right)$ in Equations (31) and (32) can be determined by the voltage- and current-output cases, respectively, in the following subsections.

### 3.2. The Special Solution and Transfer Functions

#### 3.2.1. Voltage-Output Case

In the voltage-output case, the transfer functions with ${\tilde{c}}_{\Delta }\left(z,s\right)$, ${\tilde{\varphi }}_{\Delta }\left(z,s\right)$, and $\tilde{V}\left(s\right)$ as outputs are obtained as follows [54]:

(34) $${\tilde{c}}_{\Delta }\left(z,s\right)=G_{c}\left(z,s\right)\tilde{\kappa }\left(s\right),$$

(35) $${\tilde{\varphi }}_{\Delta }\left(z,s\right)=G_{\varphi }\left(z,s\right)\tilde{\kappa }\left(s\right),$$

(36) $$\tilde{V}\left(s\right)=G_{V}\left(s\right)\tilde{\kappa }\left(s\right),$$

where

(37) $$G_{c}\left(z,s\right)=h\sinh\left(N\left(s\right)\frac{z}{h}\right){\left\{N\left(s\right)\cosh\left(N(s)\right)\right\}}^{-1}D^{-1}b,$$

(38) $$G_{\varphi }\left(z,s\right)=\frac{z_{I}F\delta^{2}h^{3}}{\varepsilon (\delta^{2}h^{2}s+d_{II})}\left[\begin{array}{cc} 1 & 0 \end{array}\right]D\left[\frac{z}{h}I_{2}-\sinh\left(N\left(s\right)\frac{z}{h}\right){\left\{N\left(s\right)\cosh\left(N(s)\right)\right\}}^{-1}\right]D^{-1}b,$$

(39) $$G_{V}\left(s\right)=\frac{2z_{I}F\delta^{2}h^{3}}{\varepsilon (\delta^{2}h^{2}s+d_{II})}\left[\begin{array}{cc} 1 & 0 \end{array}\right]D\left[I_{2}-\tanh\left(N\left(s\right)\right)N{\left(s\right)}^{-1}\right]D^{-1}b.$$

Note that the obtained open-circuit voltage-output transfer function is an irrational function of *s* as it contains a square root matrix N(s) and a hyperbolic matrix function.

#### 3.2.2. Current-Output Case

In the current-output case, the transfer functions with ${\tilde{c}}_{\Delta }\left(z,s\right)$, ${\tilde{\varphi }}_{\Delta }\left(z,s\right)$, and $\tilde{I}\left(s\right)$ as outputs are derived. Equations (23) and (24) are added to Equations (31) and (32) to determine the arbitrary functions $q_{1}\left(s\right)$ and $q_{3}\left(s\right)$ as follows:

(40) $$\frac{1}{h}\left\{N\left(s\right)-\frac{1}{\delta^{2}}\left[\begin{array}{cc} 1 & 0\\ 0 & 0 \end{array}\right]N{\left(s\right)}^{-1}\right\}\cosh\left(N(s)\right)q_{1}\left(s\right)+\frac{\varepsilon }{z_{I}F\delta^{2}h^{2}}\left[\begin{array}{c} 1\\ 0 \end{array}\right]q_{3}\left(s\right)=D^{-1}b\tilde{\kappa }\left(s\right),$$

(41) $$-\frac{z_{I}Fh}{\varepsilon }\left[\begin{array}{cc} 1 & 0 \end{array}\right]M{\left(s\right)}^{-1}\sinh\left(N(s)\right)q_{1}\left(s\right)+q_{3}\left(s\right)=0.$$

In summary, $q_{1}\left(s\right),q_{3}\left(s\right)$ is expressed as

(42) $$q_{1}\left(s\right)=h{\left\{B\left(s\right)N\left(s\right)\cosh\left(N(s)\right)\right\}}^{-1}D^{-1}b\tilde{\kappa }\left(s\right),$$

(43) $$q_{3}\left(s\right)=\frac{z_{I}Fh^{2}}{\varepsilon }\left[\begin{array}{cc} 1 & 0 \end{array}\right]M{\left(s\right)}^{-1}\tanh\left(N(s)\right){\left\{B\left(s\right)N\left(s\right)\right\}}^{-1}D^{-1}b\tilde{\kappa }\left(s\right),$$

where

(44) $$B\left(s\right):=I_{2}+\frac{1}{\delta^{2}h^{2}s+d_{II}}\left[\begin{array}{cc} 1 & 0\\ 0 & 0 \end{array}\right]D\left\{\tanh\left(N(s)\right)N{\left(s\right)}^{-1}-I_{2}\right\}.$$

Substituting Equations (42) and (43) into Equations (31) and (32), we obtain special solutions for ${\tilde{c}}_{\Delta }\left(z,s\right)$ and ${\tilde{\varphi }}_{\Delta }\left(z,s\right)$ as follows:

(45) $${\tilde{c}}_{\Delta }\left(z,s\right)=H_{c}\left(z,s\right)\tilde{\kappa }\left(s\right),$$

(46) $${\tilde{\varphi }}_{\Delta }\left(z,s\right)=H_{\varphi }\left(z,s\right)\tilde{\kappa }\left(s\right),$$

where

(47) $$H_{c}\left(z,s\right)=h\sinh\left(N\left(s\right)\frac{z}{h}\right){\left\{B\left(s\right)N\left(s\right)\cosh\left(N(s)\right)\right\}}^{-1}D^{-1}b,$$

(48) $$\begin{array}{ccc} H_{\varphi }\left(z,s\right) & = & \frac{z_{I}F\delta^{2}h^{3}}{\varepsilon (\delta^{2}h^{2}s+d_{II})}\left[\begin{array}{cc} 1 & 0 \end{array}\right]D\left[\frac{z}{h}\tanh\left(N(s)\right)-\sinh\left(N\left(s\right)\frac{z}{h}\right){\left\{\cosh\left(N(s)\right)\right\}}^{-1}\right]\\ & & \;{\left\{B\left(s\right)N\left(s\right)\right\}}^{-1}D^{-1}b. \end{array}$$

Substituting Equation (45) into Equation (26), we obtain the transfer function with short-circuit current $\tilde{I}\left(s\right)$ at the output as follows:

(49) $$\tilde{I}\left(s\right)=H_{I}\left(s\right)\tilde{\kappa }\left(s\right),$$

(50) $$H_{I}\left(s\right)=z_{I}FS\left[\begin{array}{cc} 1 & 0 \end{array}\right]D\left(I_{2}-\tanh\left(N(s)\right)N{\left(s\right)}^{-1}B{\left(s\right)}^{-1}\right)D^{-1}b.$$

Similar to the open-circuit voltage output transfer function, the resulting short-circuit current output transfer function contains a square root matrix N(s) and a hyperbolic matrix function and is therefore an irrational function.

### 3.3. Validations and Numerical Examples

#### 3.3.1. Method

In this subsection, the correctness of the derived analytical solution is verified. The model predictions of the frequency response from Equations (39) and (50) can be readily calculated using software such as MATLAB. Direct numerical simulations are performed using COMSOL Multiphysics to obtain the frequency response of the nonlinear model described in Section 2.1. The partial differential equations are directly implemented in COMSOL as mathematical expressions. Specifically, the governing Equations (1)–(8) are input into the Coefficient Form PDE interface in COMSOL. The results of the direct numerical simulations are compared with the analytical solutions. In the voltage-output case, the COMSOL model uses 200 finite elements. In the current-output case, 3875 finite elements are used, with a finer mesh near the boundaries. The applied curvature was applied as: $\kappa \left(t\right)=50\sin\omega t\left[m^{-1}\right]$. The frequency range for analysis consists of 16 points from $10^{-5}$ to $10^{10}$ [rad/s]. The simulation time length is set to 50 cycles of the input sine wave. From the time series of the input and output data, the magnitude and phase at the frequencies ω’s were obtained using frequency analysis based on the correlation method [58].

Table 3 lists the constants used in the numerical examples. The constants in Table 3 correspond to a relative humidity of 90%, a cation type of ${\mathrm{Na}}^{+}$, and the use of gold-plated Nafion N117.

#### 3.3.2. Voltage-Output Case

A numerical example of Equation (39) is provided for the voltage-output case. Equation (39) is verified by comparison with the numerical solution of the original set of nonlinear partial differential Equations (1)–(8).

**Figure 5:**
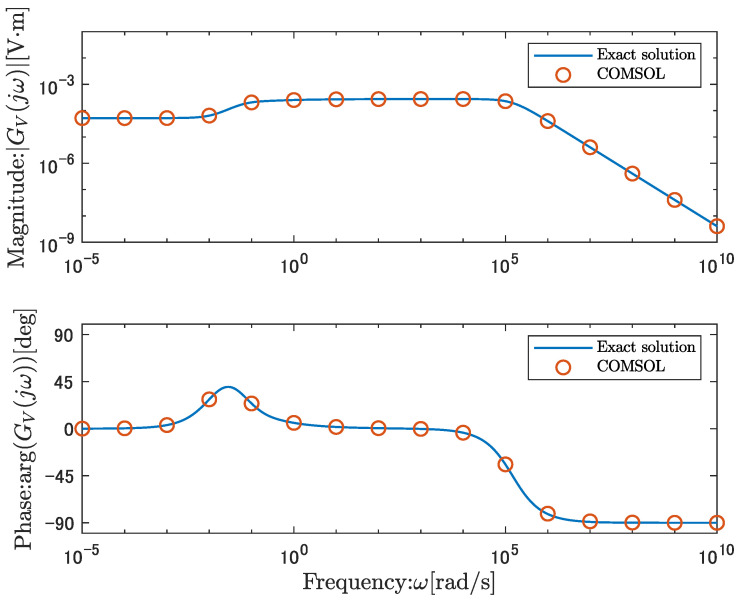
Comparison of the analytical solution, the open-circuit voltage output transfer function, $G_{V}\left(s\right)$, with the numerical solution obtained using COMSOL.

Figure 5 shows the frequency response obtained from Equation (39) as a solid line and the frequency response obtained by COMSOL as circles. Figure 5 demonstrates that the direct numerical results agree well with the analytical solution, indicating that the derived analytical solution is reasonable.

The frequency response of the analytical solution in the voltage-output case exhibits the following characteristics. Figure 5 shows that the gain becomes flat in the low-frequency range below $10^{-3}$ rad/s and has a non-zero DC gain. The gain reaches its maximum and remains nearly constant in the frequency range from $10^{-1}$ to $10^{5}$ rad/s. At frequencies above $10^{5}$ rad/s, the gain converges to zero, and no direct feedthrough term exists.

#### 3.3.3. Current-Output Case

**Figure 6:**
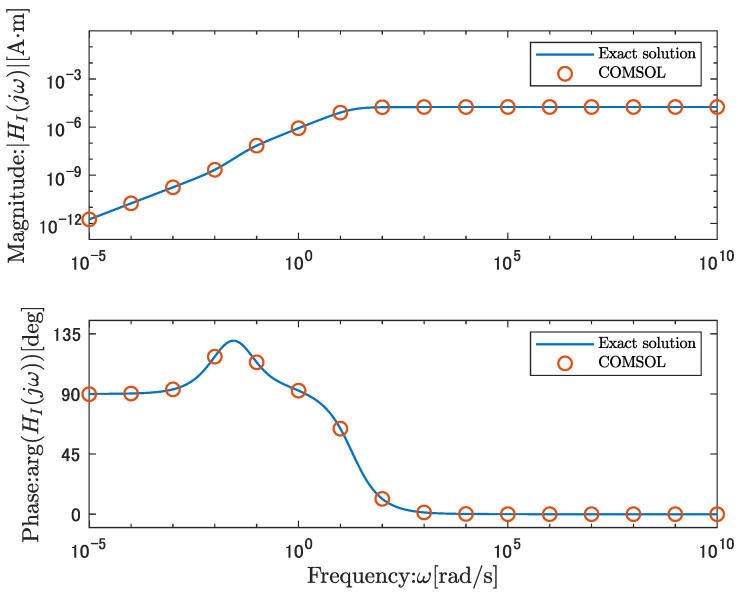
Comparison of the analytical solution, the short-circuit current output transfer function, $H_{I}\left(s\right)$, with the numerical solution obtained from COMSOL.

Similar to the voltage-output case, a numerical example of Equation (50) is provided for the current-output case. The validity of Equation (50) is confirmed by comparing it with the numerical solution of the original set of nonlinear partial differential Equations (1)–(8). Figure 6 shows the frequency response obtained from Equation (50) as a solid line and the frequency response obtained from COMSOL as circles. The direct numerical results are in good agreement with the analytical solution, indicating that the derived analytical solution is reasonable, as shown in Figure 6.

The frequency response of the analytical solution in the current-output case exhibits the following characteristics. Figure 6 shows that the DC gain is zero, which corresponds to no current flow at the frequency of zero. Equation (25) confirms that it has a DC gain of zero. The gain converges to a steady-state value at frequencies above $10^{2}$ rad/s.

## 4. Prediction of the Step Responses Using the Exact Transfer Functions

### 4.1. Predicting the Step Responses by the Approximate Transfer Functions

This section examines how variations in physical constants affect the time (step) response of the IPMC sensor using the obtained analytical solution. Deriving time-domain responses directly is challenging because the transfer functions in Equations (39) and (50) are irrational. Therefore, Equations (39) and (50) are approximated by first- or second-order rational transfer functions. The approximated transfer functions are characterized by parameters such as DC gain, direct feedthrough term, and corner frequency, which enables prediction of the sensor step response. The accuracy of the approximated step response prediction is evaluated by comparison with direct numerical simulations using COMSOL.

### 4.2. Approximations and Analysis of the Exact Transfer Functions

#### 4.2.1. Voltage-Output Case

**Figure 7:**
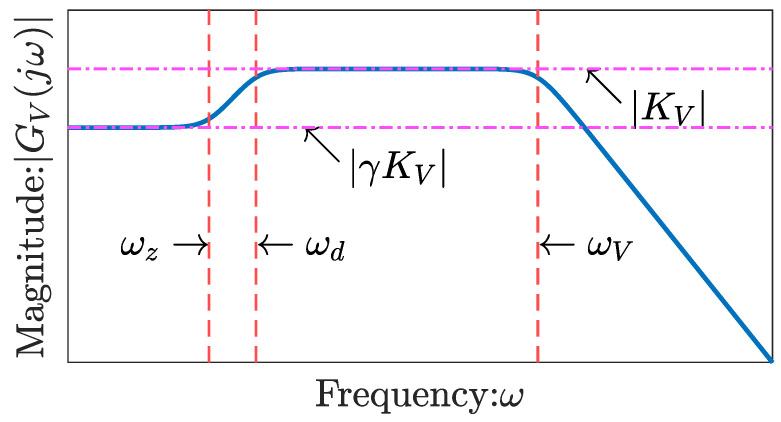
Bode diagram schematic illustrating Equation (51).

In this study, $G_{V}\left(s\right)$, provided in Equation (39), is approximated as a second-order transfer function, as follows:

(51) $$G_{V}\left(s\right)\approx K_{V}\cdot \frac{s+\omega_{z}}{s+\omega_{d}}\cdot \frac{\omega_{V}}{s+\omega_{V}},$$

where $K_{V}$ is the gain, $\omega_{z}$ and $\omega_{d}$ are the corner frequencies, and $\omega_{V}$ is the cutoff frequency. These parameters are illustrated in Figure 7. This paper presents analytical expressions for $K_{V},\omega_{V}$ and $\omega_{z}$ using physical constants. $\omega_{V}=d_{II}\delta^{-2}h^{-2}$, and the details of its derivation are provided in reference [54]. Because $\omega_{d}\ll \omega_{V}$ [54], Equation (51) can be approximated as follows:

(52) $$G_{V}\left(s\right)\approx K_{V}\frac{s+\omega_{z}}{s+\omega_{d}}.$$

$K_{V}$ is derived as follows [54,55]:

(53) $$K_{V}=\lim_{s\to \infty }G_{V}\left(s\right)\frac{\left(s+d_{II}\delta^{-2}h^{-2}\right)}{d_{II}\delta^{-2}h^{-2}}=\frac{2R_{\mathrm{gas}}ThKE_{\mathrm{wet}}}{3z_{I}Fd_{II}},$$

where $\lim_{s\to \infty }\tanh\left(N(s)\right){\left(N\left(s\right)\right)}^{-1}=O_{2}$ [54]. Subsequently, $\omega_{z}$ is derived analytically as follows [54,55]

(54) $$\begin{array}{c} \omega_{z}=\frac{G_{V}\left(0\right)}{K_{V}}\omega_{d}=\gamma \left(1-\delta \tanh\left(\delta^{-1}\right)\right)\omega_{d}, \end{array}$$

where

(55) $$\begin{array}{c} \gamma :=\frac{1}{1+\left(\frac{1-n_{dW}\frac{c_{I0}}{c_{W0}}}{1-n_{dW}\frac{d_{II}}{d_{WW}}}\right)\left(\frac{4}{9}\frac{KE_{\mathrm{dry}}}{d_{WW}}+n_{dW}\frac{d_{II}}{d_{WW}}\right)}. \end{array}$$

Because |δ|≪1, $\delta \tanh\left(\delta^{-1}\right)\approx 0$ [54,55], and Equation (54) can be approximated as follows:

(56) $$\omega_{z}\approx \gamma \omega_{d}.$$

#### 4.2.2. Current-Output Case

**Figure 8:**
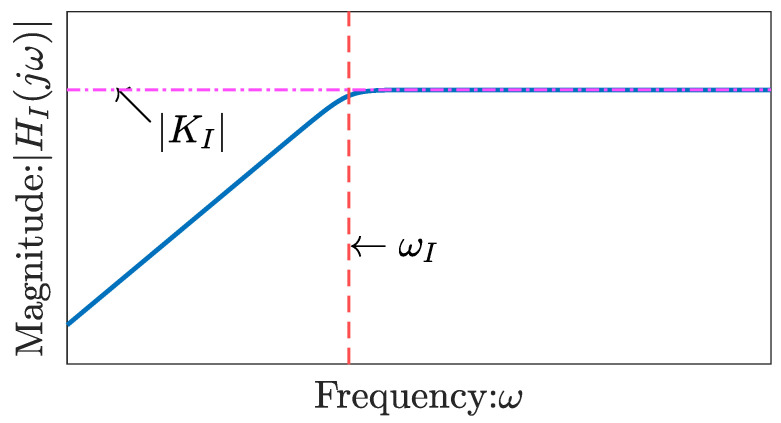
Bode diagram schematic illustrating Equation (57).

In this paper, $H_{I}\left(s\right)$, provided in Equation (50), is approximated as a first-order transfer function, as follows:

(57) $$H_{I}\left(s\right)\approx \frac{K_{I}s}{s+\omega_{I}},$$

where $K_{I}$ is the direct feedthrough term of $H_{I}$, and $\omega_{I}$ is the cutoff frequency. These parameters are illustrated in Figure 8. This paper presents analytical expressions for $K_{I}$ in terms of physical constants. Given that $K_{I}$ is the direct feedthrough term of Equation (50), $K_{I}$ is derived from Equation (50) as:

(58) $$\begin{array}{c} K_{I}=H_{I}\left(\infty \right)=\frac{z_{I}c_{I0}FSKE_{\mathrm{wet}}}{3}. \end{array}$$

### 4.3. Relationship Between the Transfer Functions (Frequency Domain) and Step Responses (Time Domain)

#### 4.3.1. Voltage-Output Case

This subsection demonstrates that Equation (52), derived in the previous subsection, is applicable for computing the time (step) response of the sensor voltage. First, the inverse Laplace transform of Equation (52) yields the step response, which is derived as:

(59) $$V\left(t\right)\approx \left\{\left(1-\gamma \right)\exp\left(-\omega_{d}t\right)+\gamma \right\}K_{V}\kappa_{\mathrm{const}}.,$$

where, $\kappa_{\mathrm{const}}.$ is the magnitude of the step input. Equation (59) shows that the step response of the voltage is characterized by three parameters: γ, $\omega_{d}$, and $K_{V}$. The magnitude of the sensor voltage is proportional to the parameter $K_{V}$. According to Equation (53), $K_{V}$ is directly proportional to four physical constants: the thickness *h*, the hydraulic permeability coefficient *K*, the elastic modulus of the wet polymer $E_{\mathrm{wet}}$, and the absolute temperature *T*. Conversely, $K_{V}$ is inversely proportional to the diffusion coefficient of the mobile ionic charge $z_{I}Fd_{II}$. Table 4 summarizes the influence of the physical constants on $K_{V}$. The relationship between the parameter γ and the physical constants is examined in detail in the following section.

**Figure 9:**
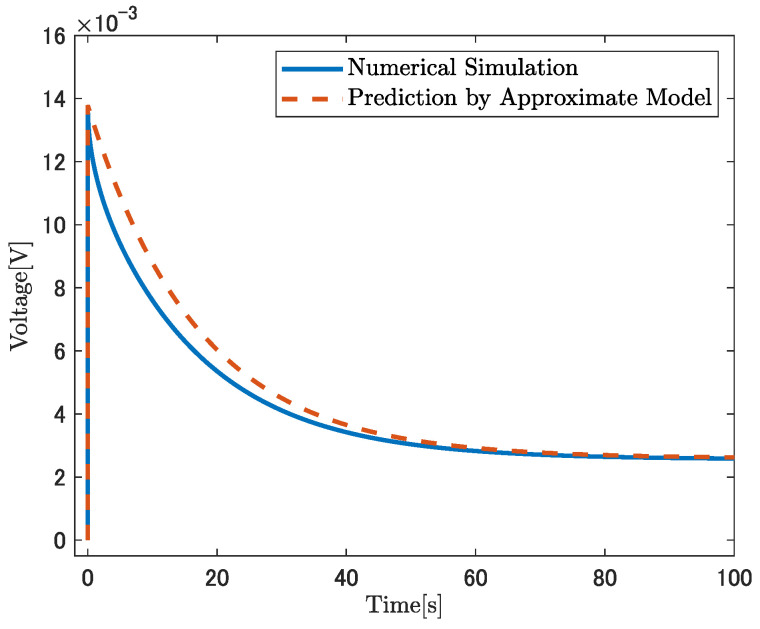
Comparison of the voltage response predicted by the approximate analytical model, Equation (59), with numerical results obtained from COMSOL.

The accuracy of Equation (59) is assessed by comparing it with a direct numerical simulation using COMSOL. The values of γ and $K_{V}$ in Equation (59) are calculated based on Table 3. The step input of $\kappa_{\mathrm{const}}.=50\;m^{-1}$ is applied. $\omega_{d}=0.0645\;\mathrm{rad}/s$ was estimated by fitting the frequency response over the range of $10^{-4}$ to 1.0 rad/s using MATLAB R2024a’s invfreqs function. The fitting is performed based on Equation (39) and the physical constants listed in Table 2. The numerical solution of the original set of nonlinear partial differential Equations (1)–(8) is used as a reference for comparison. Figure 9 shows the results of this comparison. The solid blue line represents the result of the COMSOL simulation, while the dashed red line is plotted based on Equation (59). As shown in Figure 9, the maximum and steady-state voltage values, as well as the relaxation behavior in the range from 20 to 100 s, show good agreement. This indicates that Equation (59) can reasonably approximate the step response of the IPMC sensor in the voltage-output case.

#### 4.3.2. Current-Output Case

This subsection demonstrates that Equation (57), derived in the previous subsection, is applicable for computing the time (step) response of the sensor current. Using the methodology outlined in the previous subsection, the step response of the current is derived from Equation (57) as:

(60) $$I\left(t\right)\approx \exp\left(-\omega_{I}t\right)K_{I}\kappa_{\mathrm{const}}..$$

From Equation (60), the step response of the sensor current is approximated by a single exponential function, and the peak current is characterized by $K_{I}$. According to Equation (58), $K_{I}$ is directly proportional to four (or five) physical constants: the surface area *S*, the hydraulic permeability coefficient *K*, the elastic modulus of the wet polymer $E_{\mathrm{wet}}$, and the charge density of cation $z_{I}Fc_{I0}$. Table 5 summarizes the influence of the physical constants on $K_{I}$. In contrast to the voltage-output case, the peak current does not depend on the thickness *h*.

**Figure 10:**
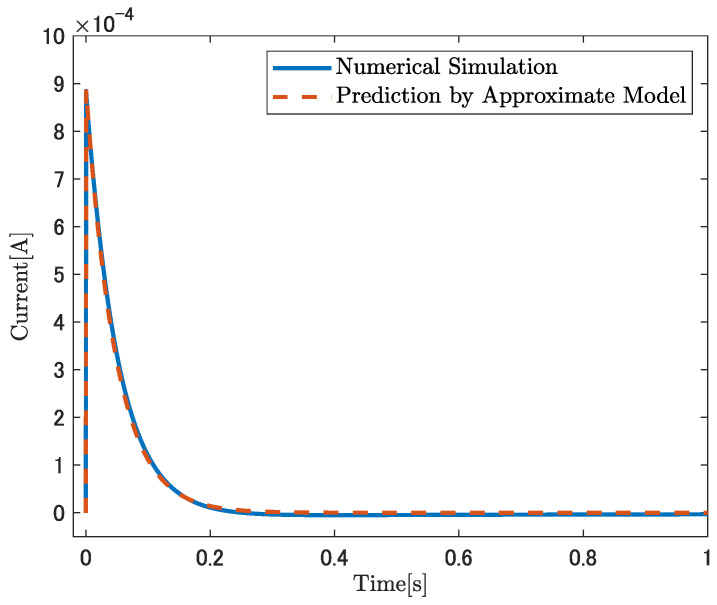
Comparison of the current response predicted by the approximate analytical model, Equation (60), with numerical results obtained from COMSOL.

The accuracy of Equation (60) is assessed by comparison with direct numerical simulations using COMSOL. The value of $K_{I}$ in Equation (60) is calculated based on Table 3. A step input of $\kappa_{\mathrm{const}}.=50\;m^{-1}$ is applied. $\omega_{I}=20.83\;\mathrm{rad}/s$ was estimated by fitting the frequency response over the range of 1.0 to $10^{4}$ rad/s using MATLAB’s invfreqs function. The fitting is performed based on Equation (50) and the physical constants listed in Table 2. The numerical solution of the original set of nonlinear partial differential Equations (1)–(8) is used as a reference for comparison. Figure 10 shows the results of this comparison. The solid blue line represents the COMSOL simulation result, and the dashed red line is obtained from Equation (60). Figure 10 shows strong agreement between the COMSOL simulation and Equation (60).

## 5. The Parameter γ and Physical Interpretation of the Relaxation Behavior of the Sensor Voltage

### 5.1. Cases of the Relaxation Behavior of the Sensor Voltage

**Figure 11:**
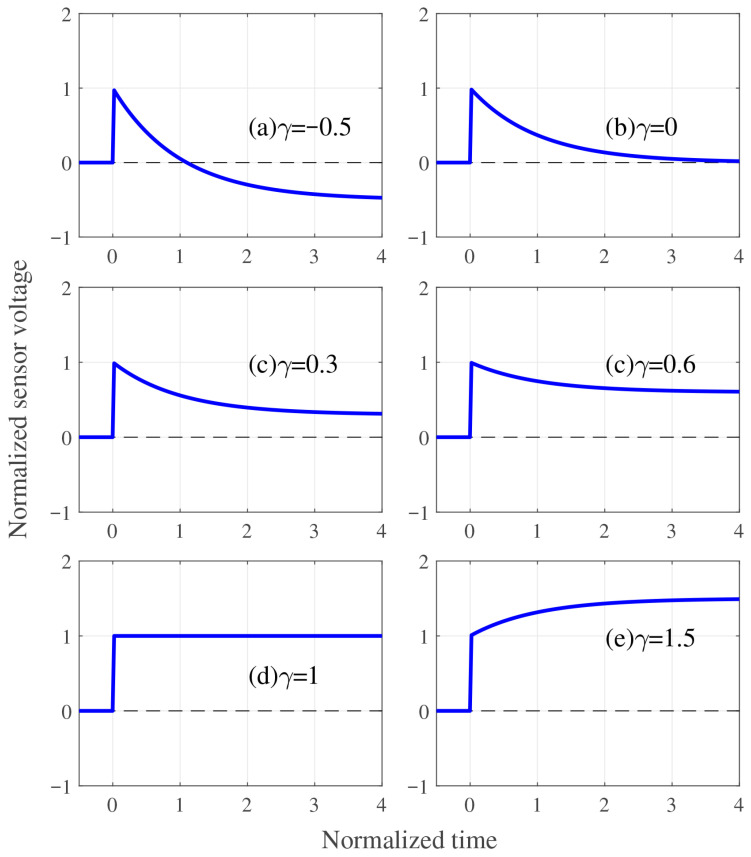
Relaxation behavior of the sensor voltage step response depending on γ.

The steady-state voltage value and relaxation behavior of the step response are described by Equation (59). It is known that certain cation types and humidity conditions induce inverse response or relaxation behavior in the voltage of an IPMC sensor [19]. Figure 11 shows a schematic of the step response of the sensor voltage, illustrating how the steady-state value and relaxation characteristics depend on γ. If γ<0, the sensor voltage decays, reaching a negative steady-state value (Case (a) of Figure 11). If 0<γ<1, the sensor voltage decays, reaching a positive steady-state value (Cases (b)–(d) of Figure 11). If γ>1, the sensor voltage monotonically increases (Case (e) of Figure 11).

Table 6 presents how the relaxation behavior, steady-state voltage sign, and maximum voltage vary with γ. The steady-state voltage values are given by $\gamma K_{V}$ and are obtained by t→∞ from Equation (59). The maximum voltage values are given by $K_{V}$ if γ≤1, and by $\gamma K_{V}$ if γ>1 from Equation (59).

The expression of γ in Equation (55) is complex due to the presence of seven physical constants; however, from Equation (55), three independent dimensionless physical constants, $KE_{\mathrm{dry}}/d_{WW}$, $n_{dW}d_{II}/d_{WW}$, and $n_{dW}c_{I0}/c_{W0}$, are identified. The cases of γ shown in Table 6 are derived from Equation (55) as follows:

(61) $$\begin{array}{ccc} \gamma <0 & ⟺ & \left(\frac{4}{9}\frac{KE_{\mathrm{dry}}}{d_{WW}}+n_{dW}\frac{d_{II}}{d_{WW}}\right)\frac{1-n_{dW}\frac{c_{I0}}{c_{W0}}}{1-n_{dW}\frac{d_{II}}{d_{WW}}}<-1, \end{array}$$

(62) $$\begin{array}{ccc} \gamma =0 & ⟺ & 1-n_{dW}\frac{d_{II}}{d_{WW}}=0, \end{array}$$

(63) $$\begin{array}{ccc} 0<\gamma <1 & ⟺ & \frac{1-n_{dW}\frac{c_{I0}}{c_{W0}}}{1-n_{dW}\frac{d_{II}}{d_{WW}}}>0, \end{array}$$

(64) $$\begin{array}{ccc} \gamma =1 & ⟺ & 1-n_{dW}\frac{c_{I0}}{c_{W0}}=0, \end{array}$$

(65) $$\begin{array}{ccc} \gamma >1 & ⟺ & -1<\left(\frac{4}{9}\frac{KE_{\mathrm{dry}}}{d_{WW}}+n_{dW}\frac{d_{II}}{d_{WW}}\right)\frac{1-n_{dW}\frac{c_{I0}}{c_{W0}}}{1-n_{dW}\frac{d_{II}}{d_{WW}}}<0. \end{array}$$

According to Equations (61)–(65), among the three dimensionless constants $KE_{\mathrm{dry}}/d_{WW}$, $n_{dW}d_{II}/d_{WW}$, and $n_{dW}c_{I0}/c_{W0}$, the latter two, $n_{dW}d_{II}/d_{WW}$ and $n_{dW}c_{I0}/c_{W0}$, play a particularly important role. In other words, the cases of γ depend on whether $n_{dW}d_{II}/d_{WW}$ or $n_{dW}c_{I0}/c_{W0}$ is greater or less than 1. If $n_{dW}d_{II}/d_{WW}<1$ and $n_{dW}c_{I0}/c_{W0}<1$, this case corresponds to 0<γ<1 in Equation (63) and (c) in Table 6, which is a common scenario for IPMC sensor responses [19,21]. If $n_{dW}d_{II}/d_{WW}>1$ and $n_{dW}c_{I0}/c_{W0}<1$, this case corresponds to γ<0 in Equation (61) and (a) in Table 6. The condition $n_{dW}d_{II}/d_{WW}>1$ indicates that the cation diffusion coefficient $d_{II}$ is very large. $n_{dW}c_{I0}/c_{W0}<1$ indicates that the equilibrium water concentration $c_{W0}$ is large. This case thus corresponds to highly mobile cations and a sufficiently wet state. If $n_{dW}d_{II}/d_{WW}<1$ and $n_{dW}c_{I0}/c_{W0}>1$, this case corresponds to γ>1 in Equation (65) and (e) in Table 6. This case arises when $d_{II}$ and $c_{W0}$ are both relatively small, indicating low cation mobility and limited swelling.

The physical meanings of the parameters $n_{dW}c_{I0}/c_{W0}$ and $n_{dW}d_{II}/d_{WW}$ are explained by Equations (3) and (4). In Equation (3), $n_{dW}c_{I0}d_{II}/c_{W0}$ is the intercoupling coefficient of $\partial c_{W}/\partial z$ with respect to the cation flux. Thus, $n_{dW}c_{I0}/c_{W0}$ represents the ratio of the intercoupling coefficient of $\partial c_{W}/\partial z$, $n_{dW}c_{I0}d_{II}/c_{W0}$, to the diffusion coefficient of cations, $d_{II}$. Similarly, in Equation (4), $n_{dW}d_{II}$ is the intercoupling coefficient of $\partial c_{I}/\partial z$ with respect to the water flux. Thus, $n_{dW}d_{II}/d_{WW}$ represents the ratio of the intercoupling coefficient of $\partial c_{I}/\partial z$, $n_{dW}d_{II}$, to the diffusion coefficient of water, $d_{WW}$.

**Figure 12:**
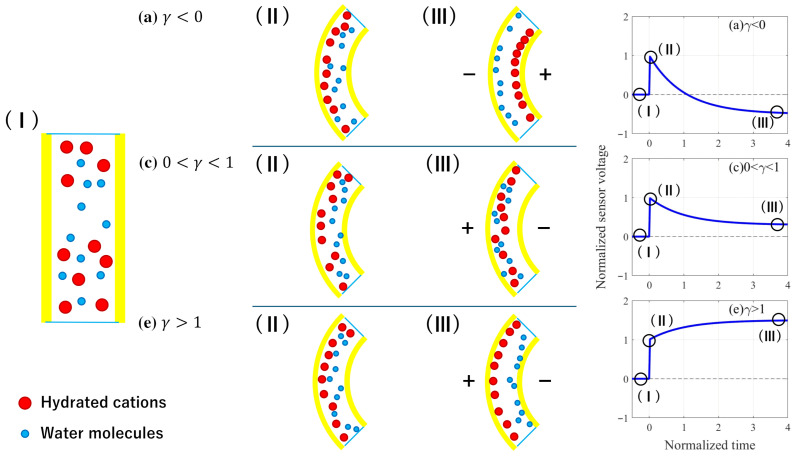
Schematic of the distribution of cations and water depending on γ. (I) Initial state; (II) state immediately after bending; and (III) steady state.

We discuss how the distribution of cations and water within an IPMC sensor varies with the value of γ. Figure 12 shows a schematic illustration of the distributions of cations and water molecules within IPMC sensors, interpreted from COMSOL simulation results. Initially, cations and water molecules are uniformly distributed within the IPMC sensor as shown in Figure 12(I). Immediately after the IPMC sensor is bent, cations move rapidly due to the pressure gradient, whereas water molecules move more slowly and do not significantly shift from their initial positions, as shown in Figure 12(II). The behavior of cations and water molecules up to this point is nearly independent of γ. However, the steady-state distributions of cations and water molecules vary considerably with the value of γ (Figure 12(III)). As a result, the sensor voltage response also varies significantly. In Figure 12(III), when 0<γ<1, the distributions of cations and water, as well as the voltage output, are consistent with physical intuition. For 0<γ<1, the steady-state distributions of cations and water exhibit higher concentrations in regions of lower pressure. However, it is difficult to intuitively understand the distributions of cations and water, as well as the voltage output, for γ<0 and γ>1. To clarify this behavior, the steady-state solutions for the cation and water molecule concentrations, $c_{\Delta }\left(z,t=\infty \right)$, is obtained. By substituting $\partial c_{\Delta }/\partial t=0$ into Equation (11), an ordinary differential equation in *z* is derived. Then, by applying Equation (18) to the resulting equation yields the following solution:

(66) $$\begin{array}{ccc} c_{\Delta }\left(z,\infty \right) & = & \left[\begin{array}{cc} \delta h\frac{\sinh\left(\delta^{-1}h^{-1}z\right)}{\cosh\left(\delta^{-1}\right)} & 0\\ 0 & z \end{array}\right]D^{-1}b\kappa_{\mathrm{const}.}. \end{array}$$

By substituting z=h into Equation (66), $c_{\Delta }$ on the boundary is obtained as follows:

(67) $$\left[\begin{array}{c} c_{I\Delta }\left(h,\infty \right)\\ c_{W\Delta }\left(h,\infty \right) \end{array}\right]=\left[\begin{array}{cc} \gamma & 0\\ 0 & \gamma \frac{1-n_{dW}\frac{d_{II}}{d_{WW}}}{1-n_{dW}\frac{c_{I0}}{c_{W0}}} \end{array}\right]\left[\begin{array}{c} \frac{c_{I0}}{d_{II}}h\delta \tanh\delta^{-1}\\ \frac{c_{W0}}{d_{WW}}h \end{array}\right]\frac{KE_{\mathrm{wet}}\kappa_{\mathrm{const}.}}{3}.\;$$

Equation (67) provides the steady-state concentrations of cations and water molecules at z=h in the IPMC sensor. According to Equation (67), if γ<0 (which corresponds to the case where $n_{dW}d_{II}/d_{WW}>1$ and $n_{dW}c_{I0}/c_{W0}<1$), then $c_{I\Delta }\left(h,\infty \right)$ is negative and $c_{W\Delta }\left(h,\infty \right)$ is positive. Therefore, the sign of the steady-state cation distribution near the boundary is opposite to the sign of the pressure. Conversely, Equation (67), if γ>1 (which corresponds to the case where $n_{dW}d_{II}/d_{WW}<1$ and $n_{dW}c_{I0}/c_{W0}>1$), then $c_{I\Delta }\left(h,\infty \right)$ is positive and $c_{W\Delta }\left(h,\infty \right)$ is negative. Therefore, the sign of the steady-state water distribution near the boundary is opposite to that of pressure.

### 5.2. Validation of the Predicted Value of γ with the Experimental Parameters

In this subsection, the predicted value of γ is compared with the experimental value of γ. The experimental value of γ is estimated from the step response shown in FIG.4 of [19]. Specifically, the experimental value of γ was calculated as the ratio of the steady-state value to the initial value of the measured step response. The predicted value of γ is calculated from Equation (55) using physical constants reported in previous studies [49,59,60,61].

Most of the physical constants can be found in previous studies but $c_{W0}$ can not. $c_{W0}$ is derived from the local volume fraction of water $w_{V}$ as shown in Equation (2) but the values of $w_{V}$ have also rarely been reported in previous studies. Therefore, $w_{V}$ is estimated from the mass fraction of water, which is more commonly reported in the literature. The mass fraction of water is defined as follows [19]:

(68) $$w_{m}=\frac{m-m_{\mathrm{dry}}}{m_{\mathrm{dry}}}=\frac{\rho_{H_{2}O}V_{w}}{\rho_{\mathrm{dry}}V_{\mathrm{dry}}},$$

where $m_{\mathrm{wet}}$ is the mass of Nafion under wet conditions, $m_{\mathrm{dry}}$ is the mass of Nafion under dry conditions, and $V_{w}$ is the local volume of water. In contrast, the volume fraction of water $w_{V}$ is defined as follows:

(69) $$w_{V}=\frac{V_{w}}{V_{s}}=\frac{V_{w}}{V_{\mathrm{wet}}-V_{w}}=\frac{1}{\frac{V_{\mathrm{wet}}}{V_{w}}-1},$$

where $V_{s}$ is the volume of the solid (which is not the same as the volume of the dry polymer), and $V_{\mathrm{wet}}$ is the volume of Nafion under wet conditions. By solving Equation (68) for $V_{w}$ and substituting the result into Equation (69), the following equation is obtained:

(70) $$\begin{array}{c} w_{V}=\frac{w_{m}}{\frac{H_{2}O}{\rho_{\mathrm{dry}}}\frac{V_{\mathrm{wet}}}{V_{\mathrm{dry}}}-w_{m}}. \end{array}$$

Assuming that Nafion is an isotropic material and that the maximum linear expansion strain due to water absorption is 0.1, the maximum value of $V_{\mathrm{wet}}/V_{\mathrm{dry}}$ is approximately 1.3 for saturated Nafion. The value of $w_{V}$ is determined by substituting the experimentally obtained $w_{m}$ into Equation (70). Furthermore, $c_{W0}$ is calculated by substituting $w_{V}$ into Equation (2), which has been solved for $c_{W0}$.

Table 7 lists the physical constants, the calculated γ values and those estimated from experimental results (FIG.4 in [19]). Table 7 presents three cases: $H^{+}$ in water, ${\mathrm{Li}}^{+}$ in water, and $K^{+}$ in RH30%. In Table 7, the numerical values of $K,E_{\mathrm{dry}},n_{dW}$, and $c^{-}$ are taken from [49], $d_{II}$ from [59], and $d_{WW}$ from [60]. The $c_{W0}$ values are calculated using Equation (70) by extracting the values of $w_{m}$ from FIG.3 of [19]. Table 7 shows that the predicted values of γ and those estimated from experimental results [19] are in reasonable agreement. The predicted values of $n_{dW}c_{I0}/c_{W0}$ and $n_{dW}d_{II}/d_{WW}$ listed in Table 7 are consistent with the discussion in the previous subsection.

## 6. Conclusions

This paper presented an exact analytical derivation of Zhu’s multiphysics model for IPMC sensors in the form of a transfer function. The input variable of the derived transfer function is the applied bending deformation, while the output variables include the open-circuit voltage or short-circuit current at the sensor terminals, as well as the distributions of cations, water molecules, and electric potential within the sensor. Moreover, this paper analytically derived the steady-state and maximum values of the step responses for the sensor voltage and current, $K_{V}$ and $K_{I}$, from the obtained transfer functions. As a result, the physical constants contributing to the maximum values of the voltage and current were clarified. Furthermore, the dimensionless parameter γ, which characterizes the sign of the steady-state value and the relaxation behavior of the voltage response, was analytically expressed for the first time in terms of physical constants. As a remaining task for future work, it is necessary to experimentally validate the accuracy of the physical constants and transfer functions derived in this study. For example, the validity of the proposed approximate solutions can be assessed by experimentally comparing the relaxation parameter γ, and the maximum values $K_{V}$ and $K_{I}$ for IPMC sensors with different thicknesses, water contents, or cation species against the theoretical predictions. Moreover, the analytical derivation of the parameters $\omega_{d}$ and $\omega_{I}$ also remains a task for future work in order to discuss the physical meaning of the time constants for voltage and current relaxation.

The solutions obtained in this study enable prediction of sensor responses without relying on computationally expensive finite element method simulations. In addition, the solutions can be applied to a range of practical situations. First, the solutions are well suited for real-time signal processing and control. While simple transfer functions are preferable for real-time control and signal processing, finite element method simulations such as those performed with COMSOL are not suitable for such purposes. In contrast, the rational transfer functions presented in Equations (52) and (57) offer a computationally efficient alternative suitable for these applications. Second, the solutions enable sensor tuning through the selection of ionic species and water content. The solutions clearly demonstrate how sensor behavior depends on physical parameters and can thus provide guidance for selecting appropriate cation species and designing encapsulation methods for specific hydration levels to achieve the desired sensor performance. Third, the solutions are expected to support material selection for sensor design. They provide a clear means of assessing how design parameters, such as polymer and electrode material choices or membrane thickness, influence sensor performance. Furthermore, the solutions are expected to contribute to the discovery and development of novel materials for IPMC sensors, particularly those designed to achieve target values of $K_{V},K_{I}$, and γ.

## Figures and Tables

**Table 1:** Variables of the IPMC multiphysics model.

Independent variables	
*t*	Time
*z*	Coordinate in the thickness direction
Dependent variables	
$J_{I}$	Cation flux
$J_{W}$	Water flux
$c_{I}$	Cation concentration
$c_{W}$	Water concentration
φ	Electric potential
$w_{V}$	Local volume fraction of water
*P*	Total pressure
κ	Curvature
*V*	Open-circuit voltage
*I*	Short-circuit current

**Table 2:** Physical constants of the IPMC multiphysics model.

Material constants	
$E_{\mathrm{dry}}$	Elastic modulus of the dry polymer
$E_{\mathrm{wet}}$	Elastic modulus of the wet polymer
*K*	Hydraulic permeability coefficient
$\varepsilon_{r}$	Effective relative dielectric constant
$z_{I}$	Valence of cation
$d_{II}$	Diffusion coefficient of cation
$\rho_{H_{2}O}$	Density of water
$M_{H_{2}O}$	Molar weight of water
$d_{WW}$	Diffusion coefficient of water
$n_{dW}$	Drag coefficient of water
$c^{-}$	Concentration of the anion fixed to the polymer
Geometric constants	
*h*	Thickness (half of the thickness)
*S*	Surface area
Universal constants	
$\varepsilon_{0}$	Permittivity of vacuum
*F*	Faraday constant
$R_{\mathrm{gas}}$	Gas constant
Environment constant	
*T*	Absolute temperature

**Table 3:** Constants used in the numerical example.

Constants	Unit	Numerical Values
Material constants		
$E_{\mathrm{dry}}$	[Pa]	$1.0\times 10^{9}$
$E_{\mathrm{wet}}$	[Pa]	$1.0\times 10^{8}$
*K*	[$m^{2}/\left(s\cdot \mathrm{Pa}\right)$]	$4.0\times 10^{-18}$
$\varepsilon_{r}$	[-]	$1.0\times 10^{5}$
$z_{I}$	[-]	1
$d_{II}$	[$m^{2}/s$]	$2.5\times 10^{-11}$
$\rho_{H_{2}O}$	[$\mathrm{kg}/m^{3}$]	$1.0\times 10^{3}$
$M_{H_{2}O}$	[kg/mol]	$1.8\times 10^{-2}$
$d_{WW}$	[$m^{2}/s$]	$1.2\times 10^{-10}$
$n_{dW}$	[-]	3
$c^{-}$	[$\mathrm{mol}/m^{3}$]	$1.3931\times 10^{3}$
$w_{V0}$	[-]	0.4779
Geometric parameters		
*h*	[m]	$1.0\times 10^{-4}$
*S*	[$m^{2}$]	$1.0\times 10^{-4}$
Universal constants		
$\varepsilon_{0}$	[F/m]	$8.8542\times 10^{-12}$
*F*	[C/mol]	$9.6486\times 10^{4}$
$R_{\mathrm{gas}}$	[J/(mol·K)]	8.314
Environment constant		
*T*	[K]	300

**Table 4:** Influence of the physical constants on the sensor voltage coefficient $K_{V}$.

Directly proportional to:	$h,K,E_{\mathrm{wet}},T$
Inversely proportional to:	$z_{I},d_{II}$

**Table 5:** Influence of the physical constants on the sensor current coefficient $K_{I}$.

Directly proportional to:	$S,K,E_{\mathrm{wet}},,z_{I},c_{I0}$

**Table 6:** Effect of γ on sensor voltage step responses. ↘ or ↗ indicates a monotonically decreasing or increasing response, respectively. + or − indicate a positive or negative value.

	(a) γ<0	(b) γ=0	(c) 0<γ<1	(d) γ=1	(e) γ>1
Relaxation behavior	↘	↘	↘	None	↗
Sign of the steady-state voltage	−	0	+	+	+
Max. voltage (per unit curvature)	$K_{V}$	$K_{V}$	$K_{V}$	$K_{V}$	$\gamma K_{V}$

**Table 7:** Physical constants for predicting the parameter γ.

Constants	Unit	$H^{+}$, Water	${\mathrm{Li}}^{+}$, Water	$K^{+}$, RH30%
$E_{\mathrm{dry}}$	[Pa]	${1.0\times 10^{8}}^{\;a}$	←	←
*K*	[$m^{2}/\left(s\cdot \mathrm{Pa}\right)$]	${4.0\times 10^{-18}}^{\;a}$	←	←
$d_{II}$	[$m^{2}/s$]	${5.3\times 10^{-10}}^{\;b}$	${1.31\times 10^{-10}}^{\;b}$	${8.6\times 10^{-11}}^{\;b}$
$d_{WW}$	[$m^{2}/s$]	${3.87\times 10^{-10}}^{\;c}$	${3.17\times 10^{-10}}^{\;c}$	${2.25\times 10^{-10}}^{\;c}$
$n_{dW}$	[-]	$2^{\;a}$	$2^{\;a}$	$1^{\;a}$
$c^{-}$	[$\mathrm{mol}/m^{3}$]	${1.39\times 10^{3}}^{\;a}$	←	←
$\rho_{H_{2}O}$/$\rho_{\mathrm{dry}}$	[-]	$0.505^{\;d}$	←	←
$V_{\mathrm{wet}}$/$V_{\mathrm{dry}}$	[-]	1.3	1.3	1.01
$w_{m}$	[-]	$0.3^{\;e}$	$0.276^{\;e}$	$0.01^{\;e}$
$c_{W0}$	[-]	$2.54\times 10^{4}$	$2.34\times 10^{4}$	$1.09\times 10^{3}$
$n_{dW}c_{I0}/c_{W0}$	[-]	0.110 (<1)	0.119 (<1)	1.28 (>1)
$n_{dW}d_{II}/d_{WW}$	[-]	2.74 (>1)	0.827 (<1)	0.382 (<1)
γ (Predicted)	[-]	−1.57	0.124	2.13
γ (Experimental)	[-]	$-1.5^{\;f}$	$0.14^{\;f}$	$2.0^{\;f}$

^a^ Table 1 in [49]. ^b^ Table 1 in [59]. ^c^ Table 1 in [60]. ^d^ In [61]. ^e^ Estimated from FIG.3 in [19]. ^f^ Estimated from FIG.4 in [19].

## Data Availability

The data that support the findings of this study are available from the corresponding author, K.T., upon reasonable request.

## Abbreviations

The following abbreviations are used in this manuscript:

IPMC	Ionic polymer metal composite
PNP	Poisson-Nernst-Planck
